# Cascading effects of geopolitical risk on environmental quality and public health in BRICS

**DOI:** 10.3389/fpubh.2026.1703933

**Published:** 2026-04-21

**Authors:** Menghao Wen, Muhammad Wasif Hanif, Muhammad Asim Afridi, Zukhruf Zahra, Shanwei Lin

**Affiliations:** 1 School of Economics and Management Hanshan Normal University, Choazhou Guangdong Province, China; 2 Department of Economics, COMSATS University Islamabad, Abbottabad, Pakistan

**Keywords:** BRICS countries, carbon emission, environmental quality, geopolitical risk, public health

## Abstract

This research investigates the critical, under-examined nexus of geopolitical risk, environmental quality (CO2 emissions), and public health in BRICS countries. Employing pooled and quantile regression estimators for panel data, the study reveals that geopolitical risk is positively associated with environmental quality, in turn, exacerbating PM2.5 concentrations. Notably, the interaction between geopolitical risk and environmental quality amplifies the negative impact on public health and life expectancy. This highlights a precarious synergy: geopolitical instability intensifies the adverse health effects of environmental pollution. The study’s novelty lies in its comprehensive, integrated approach to this complex relationship. Our findings highlight the urgent need for policymakers to adopt holistic strategies that integrate geopolitical stability with environmental and public health agendas, particularly in these rapidly emerging economies.

## Introduction

In the contemporary world, the link between geopolitical risks, environmental crises, and public health crises poses a threat to global stability. Understandings of risks—often limited to economic measures— fail to capture the broader impact that geopolitical risks can entail. Geopolitical risks are the uncertainties that derive from wars, acts of terrorism, and interstate tensions that interfere with international relations (1), and the outcome that goes beyond sectors perceived traditionally as the ones of security. There is a growing body of literature that now draws attention to the special and often undervalued capacity of geopolitical risk to worsen environmental quality, especially by increasing CO2 emissions (2–7). This degradation, in turn, aggravates capacities for public health, thus introducing a vicious cycle (4).

In the intricate web of global interconnectedness, the BRICS nations—Brazil, Russia, India, China, and South Africa—present a compelling and crucial case study. These rapidly growing economies have high footprints on the environment mainly due to burning of fossil fuels for energy production and with high incidences of geopolitical vulnerability (8). This vulnerability is worsened by the rising population concentration in densely populated urban centers exposed to the effects of aggravated environmental quality (9).

Further, major BRICS megacities are facing a pollution crisis characterized by persistently high PM2.5 concentrations—often well above WHO safe limits—alongside rising CO2 emissions and measurable adverse health outcomes (10). Hence, it allows BRICS to become a point of reference to studying the manner in which geopolitical risk translate into environmental-public health linkage on a massive scale.

While the individual effects of having geopolitical events on environmental indicators, have drawn considerable scholarly attention (2, 4, 11, 12), it is crucial to emphasize the need for a more comprehensive understanding of how geopolitical factors systematically contribute to both environmental quality and public health crises. Existing methodologies leave out the complex non-market risks associated with the geopolitical risk when evaluating environment and health outcomes. This means traditional methods fall short of counting indirect, however severe, implications of geopolitical tensions such as disrupted supply chains can lead to shortages of critical medical supplies (e.g., ventilators, pharmaceuticals) and clean energy components, forcing reliance on localized, high-emission production methods that increase carbon output (13). Such disruptions also directly impair public health system responsiveness, delaying treatments and exacerbating health crises (14, 15). Fragmented policies undermine the enforcement of transnational environmental agreements (16); for example, cooperation under the Paris Agreement may stall as nations prioritize energy security, hampering coordinated carbon-reduction efforts. Diverted resources, such as increased military spending, can crowd out public funding for renewable energy projects and environmental health monitoring, indirectly elevating emissions and vulnerability to pollution-related diseases (17, 18). This research attempts to fill this gap through a rigorous empirical investigation into cascading geopolitical risk effects into environmental quality and public health in BRICS countries. This research argues that geopolitical risk leads to increased CO2 emissions and other forms of environmental quality through various channels. The poor environmental quality then translates into pollution-related health risks and possible threats to life expectancy. The uniqueness of this research lies in its empirical investigation of relationships and its provision of a comprehensive understanding of how geopolitical risk affects public health.

This study is grounded in an integrated political economy–environment–health framework that conceptualizes geopolitical risk as a systemic macro-level shock capable of reshaping national policy priorities. Geopolitical risk—manifested through wars, interstate tensions, sanctions, and strategic rivalry alters governments’ time horizons and incentive structures, often privileging short-term security and energy concerns over long-term environmental quality. Within this framework, geopolitical risk affects public health indirectly by first influencing environmental outcomes, particularly CO2 emissions and air pollution, which subsequently translate into adverse health effects. The framework is built on three complementary strands of theory. First, political economy theory suggests that heightened geopolitical uncertainty reallocates fiscal and regulatory capacity toward defense and strategic autonomy, crowding out environmental governance (19, 20). Second, environmental economics emphasizes that weakened regulation, disrupted investment flows, and fossil-fuel lock-in increase carbon intensity and local pollution (21, 22). Third, environmental health theory establishes that deteriorating air quality—especially PM2.5 exposure—constitutes a direct and cumulative risk to population health and longevity (23, 62). Integrating these perspectives allows us to conceptualize a coherent transmission chain: geopolitical risk → environmental quality → public health deterioration. Further, this background explicitly accounts for the strength and direction of these mechanisms depending on country-specific institutional and energy-structure characteristics. In China, strong state capacity and the “dual carbon” strategy may partially buffer the emissions impact of geopolitical risk (24, 25): (25). India’s renewable energy subsidies and energy-access priorities may moderate investment-related channels (26), while Russia’s fossil-fuel-dependent growth model amplifies the military spending and energy security mechanisms (27). Brazil and South Africa face additional constraints related to political volatility and energy transition financing (28, 61). These institutional differences define key boundary conditions under which geopolitical risk translates into environmental and health outcomes.

This study makes three contributions to literature. First, it extends research on geopolitical risk by linking it to environmental and public-health outcomes rather than treating it solely as a macro-financial disturbance. Second, it develops an integrated empirical framework for examining how geopolitical instability is associated with CO2 emissions, PM2.5 exposure, and life expectancy within a common panel setting. Third, by focusing on BRICS economies and employing both pooled and quantile techniques, the study highlights heterogeneity in these relationships and offers evidence that can inform future work on geopolitics, environmental quality, and population health. Altogether, these three distinct but intertwined contributions give a more holistic and in-depth view of the cascading effects of geopolitical risk. In addition, the research presents distinct methodological approaches for sound evaluation of some of the potential socioeconomic and environmental implications. It may bridge the formerly separated areas of geopolitics, environmental economics, and public health, empirically emphasizing the urgent and immediate need to treat geopolitical stability as an underpinning of planetary health. BRICS countries face significant challenges, as the combination of rising emissions and fragile governance structures poses substantial risks to the well-being and future of millions.

These mechanisms illustrate how geopolitical risks within BRICS nations coalesce to create a pernicious self-reinforcing cycle that undermines environmental quality and public health: Through a rigorous quantitative analysis of this complex nexus, this research highlights that the enduring casualties of geopolitical conflicts and tensions may not solely appear on battlefields but rather manifest silently in the smog-choked lungs of a nation’s children and the statistical erosion of its people’s collective lifespan. In this regard, the current study aims to examine whether geopolitical risk is related to environmental quality, and whether this relationship between geopolitical risk and environmental quality is, in turn, related to health outcomes in the BRICS countries. In this regard, the current study makes an important contribution by exploring these relationships in an integrated manner.

## Literature review

The convergence of geopolitical risk, environmental quality, and public health represents a critical and underexplored nexus, particularly within the context of emerging economies (2, 4, 5, 7, 11, 12, 29–31). The BRICS countries (Brazil, Russia, India, China, and South Africa) provide an effective case study due to their rapid economic development, environmental challenges, and increasing geopolitical prominence. The prevailing literature has abundantly discussed the bilateral relationships among these variables (5, 30–33) leaving a substantial gap in the holistic understanding of their interconnected dynamics and feedback loops. A review of this literature reveals that while each element’s influence on the other has been individually investigated, a comprehensive, integrated framework for analyzing their combined effect is notably absent.

### Geopolitical risk and environmental quality

The research has shown a complex and often contradictory relationship between environmental quality and geopolitical risk. On one side, increased geopolitical risk, consisting of conflicts, trade tensions, and political instability, can directly cause destruction of the environment through military activities contaminating lands and waters, damaging ecosystems, and polluting the environment (34–38). On another hand, studies (39–41) have argued that in the short run, particularly in the short term, geopolitical risk may contribute to environmental improvement. For example, elevated geopolitical risk may discourage economic activity and industrial production, resulting in a temporary decline in CO2 emissions and other forms of pollution. This being said, these findings have a conflicting relationship that is affected by “aggregation bias”; whereas, at the panel level, a positive relation is found between geopolitical risk and environmental quality, at the disaggregated country-specific level, the opposite is observed regarding nations such as China, India, and South Africa, where geopolitical risk curtails polluting industries (37). Besides, geopolitical risk can also indirectly aggravate environmental quality by shifting national priorities (42, 43), for example, to enhance energy security, countries may increase the consumption of fossil fuels, undermine environmental quality efforts and deter international cooperation on climate change. Geopolitical rivalry is also redefining global environmental discourse, as the BRICS bloc emphasizes its stimulus and advocating climate policy through the lens of finance and social justice, sometimes pushing back against climate-related trade barriers from developed nations (44, 45, 60).

### Environmental quality and public health

The literature on the relationship between environmental quality and public health is abundant (46–48). Further, the industrialization progress and swift urbanization characterizing emerging economies have also polluted the air and water, which has severe public health implications (49). In the BRICS countries, there is a consistent and significant positive correlation between environmental quality and healthcare expenditures (37, 50). Air pollution from industrial sources, vehicular traffic, and household fuels adds to a high burden of respiratory and cardiovascular diseases (49, 51–53), while contaminated water sources lead to the spread of waterborne illnesses (54, 55). Beyond direct pollution, climate change, driven by environmental quality, is a major public health threat, displaying extreme weather events, heat-related morbidity, and the spread of vector-borne diseases (56). These health challenges place a significant strain on already-pressured public health systems in these nations. Conversely, studies have also shown that environmental conservation, such as the preservation of forest reserves, can positively impact public health by improving air and water quality and reducing the economic burden on healthcare systems.

### The missing triadic framework linking geopolitical risk, environment, and health

Although there is some evidence of these bilateral relationships, the causal pathways within the “triad” of geopolitical risk, environmental quality, and public health remain theoretically and empirically underdeveloped. A key theoretical gap is that there has been no cohesive framework describing the complex, non-linear feedback loops among the three variables. Current theories focused on resource scarcity but do not fully address the ways in which geopolitical events can change environmental policy, which ultimately leads to impact public health. For instance, a geopolitical conflict may impact supply chains, making a country turn to cheaper and more polluting energy sources, which in turn will cause an increase in respiratory illnesses. This causal chain needs to be modeled and tested with greater theoretical precision.

### Gaps in the existing evidence base

Empirically speaking, many considerable gaps exist. Most studies use aggregate panel data, which by Riti et al. (37), have noted can conceal important country-specific dynamics and lead to aggregation bias. The need now arises for more panel studies that uses data for specific regions of a group of countries associated in political or economic alliances like the BRICS countries. In addition, while health expenditures are often an alternative measure of public health impact, future studies should, in fact, use more public health indicators like life expectancy (representing how well the health system has been translated in the public health) and exposure of the public to air pollution (PM2.5, i.e., mean annual exposure in micrograms per cubic meter) to investigate the consequences on public health very specifically. Possible measures of geopolitical risk can be expanded beyond the traditional indicators of conflict and unrest to cover trade policy uncertainty, sanctions, etc., which would carry some local environmental and health impacts, but are not yet studied sufficiently. There is, therefore, a pressing need for empirical work examining how far such policies can tackle the intertwined challenges and how best to implement environmental regulation and public health measures during periods of geopolitical turbulence.

## Data and methodology

This study investigates the relationships between geopolitical risk, environmental quality, and public health in the BRICS countries (Brazil, Russia, India, China, and South Africa). The analysis spans the period from 1990 to 2023, utilizing a balanced panel dataset for all five countries of BRICS region. The choice of BRICS countries is driven because of their critical geopolitical standing, economic importance, and environmental footprints, looking for a context for examining the complex nexus under consideration. The variables employed in this study, along with their respective sources, are detailed in Table 1. The inclusion of GDP per capita, trade openness, FDI, population, and financial development helps reduce—but cannot eliminate—endogeneity concerns.

**Table 1:** Variable definitions and sources.

Indicator name	Long definition
Geopolitical risk (GPRC)	Country specific Geopolitical Risk Index developed by Caldara and Iacoviello (1), constructed for 44 developed and emerging countries including BRICS countries. It captures country referenced geopolitical events. It also reflect heterogeneous geopolitical risk profiles across countries rather than imposing a uniform global shock. A higher index value means greater geopolitical risk. https://www.matteoiacoviello.com/gpr.htm
Environmental quality (CO2 emissions, total)	Sourced from World development Indicators (59), a measure of annual emissions of carbon dioxide (CO2). This proxy of Environmental quality represent an integrative, economy-wide measure of environmental pressure arising from energy consumption, industrial activity, and land-use change. While environmental quality is inherently multidimensional, CO2 emissions remain the most widely used and policy-relevant indicator in cross-country studies examining the geopolitics–environment nexus. Country-specific environmental challenges—such as deforestation in Brazil, coal reliance in South Africa, or industrial pollution in China and India—ultimately manifest through carbon-intensive development pathways, justifying the use of CO2 as a core proxy.
$\mathrm{PM}2.5_{\mathrm{it}}\;$ air pollution, mean annual exposure (micrograms per cubic meter)	Sourced from World development Indicators (59), Population-weighted exposure to ambient PM2.5 pollution is defined as the average level of exposure of a nation’s population to concentrations of suspended particles measuring less than 2.5 microns in aerodynamic diameter, which are capable of penetrating deep into the respiratory tract and causing severe health damage.
Life expectancy at birth (LE):	From World development Indicators (59), this variable signifies the average number of years a newborn infant would live if prevailing patterns of age-specific mortality rates at the time of birth were to stay the same throughout the infant’s life. It is utilized as a primary proxy for public health outcomes.
GDP per Capita (GDPPC):	Acquired from World development Indicators (59), this variable represents GDP divided by mid-year population, expressed in constant 2015 US dollars. It serves as a control variable for economic development.
Foreign direct investment (FDI):	Sourced from World development Indicators (59), this variable indicates the net inflows of foreign direct investment as a percentage of GDP, accounting for the role of external capital in economic development and potential environmental implications.
Trade (TO):	From World development Indicators (59), this variable represents the sum of exports and imports of goods and services measured as a share of gross domestic product, serving as a control for trade openness.
Population (POP):	Total population data is drawn from World development Indicators (59), included as a demographic control variable given its influence on resource consumption and environmental pressure.
Financial development index (FD):	FD captures the depth, access, and efficiency of financial institutions and markets, controlling for the level of financial sector development. The index is sourced from www.imf.org

### Econometric model specification

Although the CO2 emissions, PM2.5 exposure, and life expectancy are dynamic processes and system GMM is appropriate for estimation. In this study, highly instrumented dynamic estimators such as system GMM are not ideal in a panel with only five countries, where instrument proliferation and weak finite-sample properties can distort inference. Therefore, to thoroughly investigate the proposed relationships, a multi-stage static econometric approach is adopted, structured to analyze the direct and indirect pathways through which geopolitical risk influences environmental quality and subsequently impacts public health.

#### First stage: geopolitical risk and environmental quality (CO2 emissions)

This stage examines the impact of geopolitical risk and other macroeconomic factors on total CO2 emissions. Three models of increasing complexity are specified to capture various influencing factors specified in Equations 1–3 to capture various:


$$\text{lnCO}2_{\mathrm{it}}=\beta_{0}+\beta_{1}\mathrm{GPR}C_{\mathrm{it}}+\beta_{2}\text{lnGDPP}C_{\mathrm{it}}+\beta_{3}\ln\mathrm{PO}P_{\mathrm{it}}+\in_{\mathrm{it}}$$ (1)

$$\begin{array}{l} \text{lnCO}2_{\mathrm{it}}=\beta_{0}+\beta_{1}\mathrm{GPR}C_{\mathrm{it}}+\beta_{2}\text{lnGDPP}C_{\mathrm{it}}\\ +\beta_{3}\text{lnPO}P_{\mathrm{it}}+\beta_{4}\mathrm{lnT}O_{\mathrm{it}}+\in_{\mathrm{it}} \end{array}$$ (2)

$$\begin{array}{l} \text{lnCO}2_{\mathrm{it}}=\beta_{0}+\beta_{1}\mathrm{GPR}C_{\mathrm{it}}+\beta_{2}\text{lnGDPP}C_{\mathrm{it}}+\beta_{3}\ln\mathrm{PO}P_{\mathrm{it}}\\ +\beta_{4}\mathrm{lnT}O_{\mathrm{it}}+\beta_{5}\text{lnFD}I_{\mathrm{it}}+\beta_{6}FD_{\mathrm{it}}+\in_{\mathrm{it}} \end{array}$$ (3)

Where *i* denotes the country and *t* denotes the year. $\epsilon_{\mathrm{it}}$ is the error term.

#### Second stage: environmental quality, geopolitical risk and health proxy (PM2.5)

This stage focuses on the immediate public health consequences of environmental quality, specifically *PM2.5* air pollution, while also considering the moderating role of geopolitical risk. Interaction terms are included to assess how geopolitical risk might amplify or mitigate the effect of *CO2* on *PM2.5*, as specified in Equations 4–6.


$$\begin{array}{l} \mathrm{PM}2.5_{\mathrm{it}}=\alpha_{0}+\alpha_{1}\text{lnCO}2_{\mathrm{it}}+\alpha_{2}\mathrm{GPR}C_{\mathrm{it}}+\alpha_{3}\mathrm{CO}2\;\text{XGPR}C_{\mathrm{it}}\\ +\alpha_{4}\text{lnPO}P_{\mathrm{it}}+\alpha_{5}\ln\text{GDPP}C_{\mathrm{it}}+\nu_{\mathrm{it}} \end{array}$$ (4)

$$\begin{array}{l} \mathrm{PM}2.5_{\mathrm{it}}=\alpha_{0}+\alpha_{1}\text{lnCO}2_{\mathrm{it}}+\alpha_{2}\mathrm{GPR}C_{\mathrm{it}}+\alpha_{3}\mathrm{CO}2\;\text{XGPR}C_{\mathrm{it}}\\ +\alpha_{4}\text{lnPO}P_{\mathrm{it}}+\alpha_{5}\ln\text{GDPP}C_{\mathrm{it}}+\alpha_{6}\mathrm{lnT}O_{\mathrm{it}}+\nu_{\mathrm{it}} \end{array}$$ (5)

$$\begin{array}{l} \mathrm{PM}2.5_{\mathrm{it}}=\alpha_{0}+\alpha_{1}\text{lnCO}2_{\mathrm{it}}+\alpha_{2}\mathrm{GPR}C_{\mathrm{it}}+\alpha_{3}\mathrm{CO}2\;\text{XGPR}C_{\mathrm{it}}\\ +\alpha_{4}\text{lnPO}P_{\mathrm{it}}+\alpha_{5}\ln\text{GDPP}C_{\mathrm{it}}+\alpha_{6}\mathrm{lnT}O_{\mathrm{it}}\\ +\alpha_{7}\text{lnFD}I_{\mathrm{it}}+\alpha_{8}FD_{\mathrm{it}}+\nu_{\mathrm{it}} \end{array}$$ (6)

Where, $\mathrm{PM}2.5_{\mathrm{it}}$ represents Air Pollution. ( $\mathrm{CO}2\;\text{XGPR}C_{\mathrm{it}}$ ) is an interaction term to capture the combined effect of *CO2* emissions and geopolitical risk on *PM2.5*. $\nu_{\mathrm{it}}$ is the error term.

#### Third stage (robustness check): environmental quality, geopolitical risk and health proxy life expectancy

For robustness check for the public health dimension, the study examines the impact of CO2 emissions and geopolitical risk on life expectancy. This stage helps to validate the findings from the *PM2.5* models and offers a broader perspective on health outcomes life expectancy, as specified in Equations 7–9.


$$\begin{array}{l} LE_{\mathrm{it}}=\alpha_{0}+\alpha_{1}\text{lnCO}2_{\mathrm{it}}+\alpha_{2}\mathrm{GPR}C_{\mathrm{it}}+\alpha_{3}\mathrm{CO}2\;\text{XGPR}C_{\mathrm{it}}\\ +\alpha_{4}\text{lnPO}P_{\mathrm{it}}+\alpha_{5}\ln\text{GDPP}C_{\mathrm{it}}+\eta_{\mathrm{it}} \end{array}$$ (7)

$$\begin{array}{l} LE_{\mathrm{it}}=\alpha_{0}+\alpha_{1}\text{lnCO}2_{\mathrm{it}}+\alpha_{2}\mathrm{GPR}C_{\mathrm{it}}+\alpha_{3}\mathrm{CO}2\;\text{XGPR}C_{\mathrm{it}}\;\\ +\alpha_{4}\text{lnPO}P_{\mathrm{it}}+\alpha_{5}\ln\text{GDPP}C_{\mathrm{it}}+\alpha_{6}\mathrm{lnT}O_{\mathrm{it}}+\eta_{\mathrm{it}} \end{array}$$ (8)

$$\begin{array}{l} LE_{\mathrm{it}}=\alpha_{0}+\alpha_{1}\text{lnCO}2_{\mathrm{it}}+\alpha_{2}\mathrm{GPR}C_{\mathrm{it}}+\alpha_{3}\mathrm{CO}2\;\text{XGPR}C_{\mathrm{it}}\\ +\alpha_{4}\text{lnPO}P_{\mathrm{it}}+\alpha_{5}\ln\text{GDPP}C_{\mathrm{it}}+\alpha_{6}\mathrm{lnT}O_{\mathrm{it}}+\alpha_{7}\text{lnFD}I_{\mathrm{it}}\\ +\alpha_{8}FD_{\mathrm{it}}+\eta_{\mathrm{it}} \end{array}$$ (9)

Where *LE* denotes Life Expectancy at Birth. $\eta_{\mathrm{it}}$ is the error term.

### Econometric techniques

This study employs a variety of econometric techniques specifically selected to correspond to the structure of the panel dataset, which encompasses five cross-sectional units (N = 5) observed over duration (t = 34). This specification is intentionally parsimonious because of the very small cross-section (N = 5), the longtime dimension (t = 34), and the need for a balanced panel over 1990–2023. The first operation is the application of the Pooled Ordinary Least Squares (OLS) method, which essentially treats all observations as independent, providing baseline estimates that provide a first, general view of the relationships among the other variables. Though it does not account for unobserved differences across countries or possible correlation over time within countries (57), it does anchor results for comparison from the more advanced estimation techniques. Subsequently, to capture variations in the effects of the explanatory variables at different points in the dependent variables’ distribution, it will then apply the quantile regression estimator for panel data (QRPD). This technique is not used to exploit cross-sectional heterogeneity per se, but rather to uncover distributional heterogeneity over time within countries, which is appropriate for long panels (T ≫ N) as shown by Powell (58). This method estimates reduced-form conditional relationships rather than structural causal parameters. It also effectively handles outliers and provides more comprehensive information by estimating effects across multiple quantiles, such as the 10th, 25th, 50th, 75th, and 90th percentiles. QRPD maintains a non-separable error term which usually correlates with quantile estimation. It estimates the effect of exogenous or endogenous independent variables on dependent variables using “within” variation in the instrument of identification purposes. Most quantile panel data estimators include additive fixed effects which separates the error term and assumes the parameters change with respect to time. Hence, QRPD is employed to explore how geopolitical risk influences environmental quality and then how geopolitical risk, and environmental quality explain public health indicated by PM2.5 and life expectancy. This method is suitable given the time-varying conditions and environmental challenges faced by the BRICS countries, allowing a robust understanding distinct from what the commonly used mean-based regression models provide.

### Testing for cross sectional dependency and reverse causality

The Pesaran (63) cross-sectional dependence test indicates that cross-sectional dependence is not statistically significant among BRICS countries (CD = 0.900, *p* = 0.368), suggesting that common shocks do not dominate the panel dynamics and that the use of Dumitrescu–Hurlin (DH) causality test is appropriate for checking reverse causality. DH causality results reveal a unidirectional causal relationship from geopolitical risk (GPRC) to CO2 emissions (lnCO2). The standard Z-bar statistics are significant (Z = 5.109, *p* = 0.000). Conversely, CO2 emissions do not Granger-cause geopolitical risk (Z = 1.844, *p* = 0.890), confirming the absence of reverse causality. The results are presented in Appendix B. These findings demonstrate that, at the panel level, reverse causality from emissions to geopolitical risk is not present, mitigating concerns regarding this source of endogeneity. Further, Sectional Dependency and Reverse Causality” that the DH test is used only as a limited diagnostic on temporal predictability and reverse causation, not as proof of exogeneity or causal identification.

## Results and analysis

The descriptive statistics for the BRICS nations from 1990 to 2023 reveal a heterogeneous group with varied performances across environmental, public health, geopolitical, and economic dimensions. Regarding environmental quality, reflected by CO2 emissions, the panel mean is 2,289.270 units, but individual country averages show a vast disparity. China stands out with the highest average emissions at 7,269.482, indicating its substantial environmental footprint, while Brazil and South Africa maintain comparatively lower averages (390.784 and 404.151, respectively). This wide range (226.895 to 13,259.640) and high standard deviation (3,071.986) underscore diverse environmental policies and industrialization trajectories.

Public health, captured by PM2.5 air pollution, averages 33.011 micrograms per cubic meter across the panel. India faces the most significant challenge, with an average PM2.5 of 61.672, followed by China at 47.609. Brazil, conversely, reports a much lower average of 14.441. The considerable standard deviation (19.125) and range (11.273 to 79.037) highlight the uneven burden of air pollution-related health issues. Life expectancy (LE), averaging 68.052 years, shows China and Brazil leading with 74.116 and 71.696 years, respectively, while South Africa trails at 60.681. The smaller standard deviation (5.839) in LE suggests some convergence in health longevity despite environmental differences. It is important to note that LE is influenced by a wide range of factors beyond those modeled here, including healthcare access, education, nutrition, and regional health policies. While our analysis focuses on the roles of geopolitical risk and environmental quality, other determinants also shape population health outcomes.

Geopolitical risk (GPRC) averages 0.322, but Russia’s significantly higher average (0.822) contrasts sharply with Brazil (0.049) and South Africa (0.065), pointing to substantial variations in political stability and exposure to geopolitical events. The broad range (0.018 to 3.690) highlights the inherent volatility. GDP per capita (GDPPC) averages 5,401.098, with Brazil (7,747.889) and Russia (7,701.764) leading, and India lower at 1,152.809. The massive standard deviation (3,082.894) reflects the diverse economic development stages (see Table 2).

**Table 2:** Descriptive statistics (panel-wise) and overall sample.

Variable	CO2	PM25	LE	GPRC	GDPPC	POP	FDI	TO	FD
Country averages									
Brazil	390.784	14.441	71.696	0.049	7,747.889	185.000	2.702	24.623	0.505
Russia	1,831.037	17.241	68.378	0.822	7,701.764	145.000	1.530	53.003	0.473
India	1,550.894	61.672	65.390	0.206	1,152.809	1,170.000	1.252	36.439	0.437
China	7,269.482	47.609	74.116	0.468	5,073.229	1,300.000	3.114	41.794	0.481
SA	404.151	24.095	60.681	0.065	5,329.799	51.200	1.396	49.849	0.454
Overall sample statistics									
Mean	2,289.270	33.011	68.052	0.322	5,401.098	571.000	1.999	41.142	0.470
Std. dev.	3,071.986	19.125	5.839	0.412	3,082.894	556.000	1.584	14.560	0.106
Min	226.895	11.273	53.914	0.018	531.898	40.000	(1.756)	15.156	0.205
Max	13,259.640	79.037	78.202	3.690	12,175.200	1,440.000	9.660	110.577	0.674

The sheer scale of population (POP) is evident, with China and India averaging 1,300.000 and 1,170.000 (in millions), respectively. Foreign Direct Investment (FDI) averages 1.999%, with China attracting the most (3.114%) and a noteworthy negative minimum (−1.756%) suggesting occasional capital outflows. Trade openness (TO) averages 41.142%, with Russia (53.003%) and South Africa (49.849%) being the most integrated, while Brazil (24.623%) is less so. Finally, Financial development (FD), averaging 0.470, shows a tighter cluster among nations (ranging from 0.205 to 0.674), indicating a more consistent level of financial sector maturity across the bloc. These statistics collectively emphasize the intricate and divergent paths of BRICS nations in navigating environmental and public health challenges amidst varying geopolitical and economic landscapes.

### Correlation analysis

The correlation matrix (see Table 3) reveals several compelling relationships among geopolitical risk, environmental quality, public health, and key socioeconomic indicators within the BRICS economies. A strong positive correlation (0.872***) exists between CO2 emissions (environmental quality) and the interaction term CO2GPRC (CO2 * Geopolitical Risk), suggesting that geopolitical risk significantly amplifies the impact of CO2 on other factors or that its influence is strongly tied to emissions. Interestingly, CO2 also exhibits a moderate positive correlation with PM2.5 (public health indicator) at 0.344**, indicating that higher CO2 emissions are generally associated with poorer air quality. Furthermore, CO2 shows a substantial positive correlation with population (POP) (0.652***), highlighting the environmental pressure exerted by large populations. PM2.5, as a direct public health indicator, displays a very strong positive correlation with population (POP) (0.875***), underscoring the severe air quality challenges in densely populated BRICS regions. Conversely, PM2.5 has a strong negative correlation with GDP per capita (GDPPC) (−0.778***), implying that economic prosperity is significantly associated with improved air quality. This suggests that as nations develop, they may invest more in pollution control or shift towards cleaner industries.

**Table 3:** Correlation matrix.

Variables	CO2	PM25	LE	GPRC	CO2GPRC	GDPPC	FDI	TO	FD	POP
CO2	1									
PM25	0.344**	1								
LE	0.596***	−0.022	1							
GPRC	0.373***	−0.093	0.306***	1						
CO2GPRC	0.872***	0.111	0.522***	0.611***	1					
GDPPC	0.200***	−0.778***	0.445***	0.336***	0.398***	1				
FDI	0.136*	−0.027	0.378***	−0.228***	−0.061	0.080	1			
TO	0.142*	−0.032	−0.122	0.230***	0.091	0.059	0.024	1		
FD	0.326***	−0.225***	0.507***	0.133*	0.361***	0.599***	0.259***	0.199***	1	
POP	0.652***	0.875***	0.381***	0.071	0.422***	−0.500***	0.152**	−0.082	0.011	1

***p < 0.01, **p < 0.05, *p < 0.1.

Life expectancy (LE) shows a robust positive correlation with CO2 (0.596***). While this finding initially appears counterintuitive from an environmental health perspective, it likely reflects the complex, early-stage industrial development pathways, where initial increases in energy consumption (and associated CO2 emissions) coincide with broader improvements in living standards, healthcare infrastructure, and food security, all of which contribute to rising life expectancy. This underscores the multifaceted drivers of life expectancy, many of which fall outside the direct scope of this study. LE is also positively correlated with GDPPC (0.445***) and Financial Development (FD) (0.507***), reinforcing the well-known link between economic advancement, financial access, and better health outcomes.

Geopolitical risk (GPRC) exhibits a moderate positive correlation with CO2 (0.373***) and a weaker one with PM2.5 (−0.093), although this is not statistically significant at conventional levels. Its positive correlation with GDPPC (0.336***) and Trade Openness (TO) (0.230***) suggests that even economically dynamic and open economies can face considerable geopolitical instability.

Overall, the correlation matrix paints a picture of intricate interdependence. While economic development and financial advancement generally correlate with improved public health (lower PM2.5, higher LE), these benefits are often accompanied by increased CO2 emissions, particularly in large, growing populations. Geopolitical risk emerges as a significant, though complex, factor affecting environmental trajectories, suggesting it either directly contributes to emissions or modulates the relationship between other variables and environmental outcomes. This preliminary analysis highlights the need for more sophisticated econometric modeling to untangle these complex causal pathways.

### Initial regression analysis

The regression analysis, encompassing both pooled OLS and generalized quantile regression (GQR) for *lnCO2* (environmental quality), offers a subtle understanding of the factors influencing CO2 emissions in BRICS countries. The pooled OLS results consistently show that geopolitical risk, population, and per capita income all exert a positive and statistically significant impact on CO2 emissions. This suggests that increased geopolitical instability, population growth, and economic expansion are associated with a rise in CO2 emissions. Notably, trade openness, when introduced, also significantly and positively contributes to CO2 emissions. Conversely, financial development emerges as a crucial mitigating factor, demonstrating a strong negative association with CO2 emissions in the most comprehensive pooled model, implying that a robust financial sector can facilitate cleaner development pathways. The high R-squared values across these pooled models (0.761 to 0.917) indicate their substantial explanatory power. The pooled regression results are depicted in Table 4.

**Table 4:** Pooled regression model (dep var.: CO2 emissions).

GPRC	0.900***	0.948***	0.826***
	(0.111)	(0.113)	(0.107)
LNPOP	0.754***	0.736***	0.821***
	(0.042)	(0.034)	(0.036)
LNGDPPC	0.427***	0.305***	0.482***
	(0.064)	(0.048)	(0.056)
LNFDI		0.245***	0.208***
		(0.065)	(0.061)
LNTO		1.021***	1.139***
		(0.079)	(0.077)
FD			−1.652***
			(0.322)
CONSTANT	−11.453***	−14.127***	−16.824***
	(1.205)	(1.026)	(1.089)
Observations	170	170	170
R-squared	0.761	0.904	0.917

Standard errors in parentheses.

***p < 0.01.

The quantile regression estimator for panel data (QRPD), however, provides a more granular perspective by examining these relationships across different quantiles of CO2 emissions, revealing heterogeneous effects not captured by pooled OLS. The results are provided in Table 5. For instance, the impact of geopolitical risk on CO2 emissions varies across distribution. While consistently positive and significant, its coefficient is particularly pronounced at lower quantiles (e.g., 1.559 at Q-0.25) when controlling only population and income, suggesting that geopolitical risk has a stronger amplifying effect on emissions in countries or periods with relatively lower carbon footprints. When more variables like FDI and trade are included, the impact of geopolitical risk remains strong and positive across all quantiles, indicating its pervasive influence regardless of the emission level.

**Table 5:** Quantile regression estimators: results (dep var.: CO2 emissions).

Variables	Q-0.10	Q-0.25	Q-0.50	Q-0.75	Q-0.90
Equation 1					
GPRC	0.510***	1.559***	0.698***	0.885***	0.967***
	(0.092)	(0.260)	(0.194)	(0.078)	(0.041)
LNPOP	1.300***	0.853***	0.740***	0.810***	0.816***
	(0.035)	(0.099)	(0.074)	(0.030)	(0.015)
LNGDPPC	0.610***	0.368**	0.677***	0.658***	0.583***
	(0.053)	(0.149)	(0.112)	(0.045)	(0.023)
CONSTANT	−24.304***	−13.576***	−13.022***	−14.051***	−13.462***
	(1.004)	(2.831)	(2.116)	(0.854)	(0.442)
OBSERVATIONS	170	170	170	170	170
Equation 2					
GPRC	0.967***	1.006***	0.959***	1.251***	1.038***
	(0.041)	(0.211)	(0.200)	(0.142)	(0.151)
LNPOP	0.816***	0.518***	0.652***	0.648***	0.795***
	(0.015)	(0.063)	(0.060)	(0.042)	(0.045)
LNGDPPC	0.583***	0.065	0.225***	0.220***	0.321***
	(0.023)	(0.090)	(0.086)	(0.061)	(0.065)
LNFDI		0.322***	0.217*	0.126	0.052
		(0.123)	(0.116)	(0.082)	(0.088)
LNTO		0.877***	1.032***	1.005***	1.114***
		(0.148)	(0.140)	(0.099)	(0.106)
CONSTANT	−13.462***	−7.958***	−12.055***	−11.580***	−15.297***
	(0.442)	(1.926)	(1.825)	(1.291)	(1.375)
OBSERVATIONS	170	170	170	170	170
Equation 3					
GPRC	0.906***	0.863***	0.888***	0.763***	0.621***
	(0.187)	(0.173)	(0.169)	(0.122)	(0.134)
LNPOP	0.655***	0.704***	0.715***	0.905***	0.934***
	(0.062)	(0.057)	(0.056)	(0.040)	(0.044)
LNGDPPC	0.298***	0.359***	0.343***	0.567***	0.690***
	(0.098)	(0.091)	(0.089)	(0.064)	(0.070)
LNFDI	0.290***	0.294***	0.158	−0.039	−0.003
	(0.106)	(0.099)	(0.096)	(0.069)	(0.076)
LNTO	1.099***	1.152***	1.147***	1.161***	0.915***
	(0.133)	(0.124)	(0.121)	(0.087)	(0.096)
FD	−1.485***	−1.760***	−1.556***	−1.647***	−1.751***
	(0.559)	(0.519)	(0.505)	(0.365)	(0.401)
CONSTANT	−12.543***	−13.881***	−13.641***	−18.685***	−19.192***
	(1.894)	(1.756)	(1.712)	(1.236)	(1.358)
OBSERVATIONS	170	170	170	170	170

Standard errors in parentheses.

***p < 0.01, **p < 0.05, *p < 0.1.

Similarly, the positive influence of population and per capita income on CO2 emissions is consistently significant across most quantiles in the QRPD models, reinforcing their fundamental roles as drivers of environmental quality. The impact of trade openness also remains robustly positive and significant across nearly all quantiles, highlighting the consistent pressure international trade places on environmental quality. Interestingly, FDI shows a positive and significant impact on CO2 emissions at lower quantiles but becomes less significant or even slightly negative at higher quantiles in the most comprehensive QRPD model, suggesting a complex, non-linear relationship.

Crucially, financial development consistently exhibits a negative and highly significant effect on CO2 emissions across all quantiles, mirroring the pooled OLS findings (See Table 6). This reinforces the notion that a developed financial sector plays a pivotal role in promoting environmental quality, regardless of the country’s emission intensity. This consistent negative impact across the entire distribution underscores its potential as a broad-based policy lever.

**Table 6:** Pooled regression results (dep var.: PM2.5 and life expectancy).

Variables	Dep Var: PM2.5			Dep Var: life expectancy		
LNCO2	0.289***	0.250***	0.224***	−0.017***	−0.017*	−0.016*
	(0.027)	(0.040)	(0.044)	(0.005)	(0.009)	(0.010)
GPRC	−0.499***	−0.741***	−0.739***	0.036***	0.046***	0.046***
	(0.047)	(0.061)	(0.061)	(0.009)	(0.013)	(0.013)
CO2GPRC	0.001***	0.001***	0.001***	−0.001***	−0.001***	−0.001***
	(0.0001)	(0.0001)	(0.0001)	(0.0001)	(0.0001)	(0.0001)
LNPOP	−0.102***	0.003	0.031	0.095***	0.092***	0.091***
	(0.025)	(0.030)	(0.036)	(0.005)	(0.007)	(0.008)
LNGDPPC	−0.617***	−0.515***	−0.486***	0.118***	0.114***	0.113***
	(0.027)	(0.027)	(0.034)	(0.005)	(0.006)	(0.007)
LNFDI		−0.197***	−0.194***		0.008	0.008
		(0.033)	(0.033)		(0.007)	(0.007)
LNTO		0.223***	0.267***		−0.006	−0.008
		(0.053)	(0.061)		(0.012)	(0.013)
FD			−0.248			0.008
			(0.172)			(0.038)
Constant	8.469***	5.358***	4.702***	1.501***	1.602***	1.624***
	(0.576)	(0.701)	(0.834)	(0.108)	(0.152)	(0.182)
Observations	170	170	170	170	170	170
R-squared	0.907	0.931	0.932	0.851	0.852	0.852

Standard errors in parentheses.

***p < 0.01, *p < 0.1.

In summary, while pooled OLS provides an average view, the QRPD analysis reveals important conditional effects. Geopolitical risk, population, and economic growth are consistent drivers of CO2 emissions, with geopolitical risk potentially having a more pronounced amplifying effect at lower emission levels. Trade openness also consistently contributes to increased carbon footprint. Conversely, financial development consistently emerges as a significant factor in mitigating CO2 emissions across all observed emission levels, highlighting its critical role in fostering environmental quality within the BRICS context.

### Impact of CO2 emissions and geopolitical risk on public health

Having established that heightened geopolitical risk is associated with increased CO2 emissions, the subsequent analysis examines how both geopolitical risk and CO2 emissions, independently and interactively, contribute to the threat to public health, specifically measured by PM2.5 air pollution. This two-pronged threat suggests that geopolitical risk not only exacerbates environmental quality but also directly and indirectly compromises public health through atmospheric pollution. In essence, geopolitical frictions can lead to policies or circumstances that prioritize short-term gains or security over environmental safeguards, thereby increasing CO2 emissions. These elevated CO2 emissions, in turn, directly contribute to PM2.5. Concurrently, geopolitical tensions might disrupt supply chains for cleaner technologies, divert resources from environmental monitoring, or impede international cooperation on air quality initiatives, thus worsening PM2.5 levels. Therefore, the presence of both factors creates a compounding challenge for public health, as populations are exposed to the direct effects of a degraded environment alongside the systemic vulnerabilities induced by geopolitical instability.

The regression results in Table 7 show the impact of geopolitical risk and CO2 emissions on PM2.5 (indicating a public health threat), illuminating these complex interactions. The pooled OLS regression results consistently demonstrate high explanatory power, with R-squared values ranging from 0.907 to 0.932, suggesting that the chosen variables effectively capture variations in PM2.5 levels. Across all pooled specifications, CO2 emissions show a positive and highly significant impact on PM2.5, with coefficients ranging from 0.224 to 0.289. This directly confirms that poorer environmental quality, as reflected by higher CO2 emissions, is strongly associated with increased air pollution, posing a direct threat to public health.

**Table 7:** Quantile regression estimators: results (Dep var.: PM2.5).

	Q-0.10	Q-0.25	Q-0.50	Q-0.75	Q-0.90
Equation 4					
LNCO2	0.218***	0.229***	0.410***	0.420***	0.454***
	(0.028)	(0.039)	(0.032)	(0.035)	(0.034)
GPRC	−0.676***	−0.713***	−0.866***	−0.589***	−0.302***
	(0.049)	(0.068)	(0.055)	(0.061)	(0.058)
CO2GPRC	0.000***	0.000***	0.000***	0.000***	0.000
	(0.000)	(0.000)	(0.000)	(0.000)	(0.000)
LNPOP	−0.206***	−0.161***	−0.157***	−0.159***	−0.140***
	(0.026)	(0.036)	(0.029)	(0.032)	(0.031)
LNGDPPC	−0.685***	−0.650***	−0.610***	−0.614***	−0.609***
	(0.028)	(0.039)	(0.032)	(0.035)	(0.033)
CONSTANT	11.342***	10.213***	8.772***	8.800***	8.218***
	(0.595)	(0.830)	(0.670)	(0.739)	(0.708)
Equation 5					
LNCO2	0.069	0.186***	0.261***	0.313***	0.460***
	(0.050)	(0.055)	(0.048)	(0.051)	(0.056)
GPRC	−0.624***	−0.927***	−0.922***	−0.688***	−0.511***
	(0.077)	(0.084)	(0.073)	(0.078)	(0.085)
CO2GPRC	0.000***	0.000***	0.000***	0.000***	0.000
	(0.000)	(0.000)	(0.000)	(0.000)	(0.000)
LNPOP	−0.052	0.027	0.004	−0.038	−0.112***
	(0.038)	(0.041)	(0.036)	(0.039)	(0.042)
LNGDPPC	−0.648***	−0.512***	−0.495***	−0.509***	−0.541***
	(0.034)	(0.037)	(0.032)	(0.034)	(0.038)
LNFDI	−0.044	−0.203***	−0.228***	−0.172***	−0.099**
	(0.041)	(0.045)	(0.039)	(0.042)	(0.046)
LNTO	0.340***	0.358***	0.264***	0.185***	0.081
	(0.067)	(0.073)	(0.064)	(0.068)	(0.074)
CONSTANT	7.926***	4.773***	5.031***	5.861***	6.922***
	(0.879)	(0.956)	(0.837)	(0.890)	(0.976)
Equation 6					
LNCO2	−0.011	0.137**	0.250***	0.307***	0.431***
	(0.052)	(0.062)	(0.049)	(0.066)	(0.060)
GPRC	−0.567***	−0.887***	−0.952***	−0.684***	−0.412***
	(0.072)	(0.086)	(0.068)	(0.092)	(0.083)
CO2GPRC	0.000***	0.000***	0.000***	0.000***	0.000
	(0.000)	(0.000)	(0.000)	(0.000)	(0.000)
LNPOP	0.009	0.046	0.023	−0.034	−0.075
	(0.042)	(0.051)	(0.040)	(0.054)	(0.049)
LNGDPPC	−0.599***	−0.501***	−0.473***	−0.508***	−0.492***
	(0.040)	(0.048)	(0.038)	(0.051)	(0.046)
LNFDI	0.005	−0.165***	−0.226***	−0.169***	−0.060
	(0.038)	(0.046)	(0.036)	(0.049)	(0.044)
LNTO	0.400***	0.413***	0.342***	0.200**	0.153*
	(0.072)	(0.086)	(0.068)	(0.091)	(0.083)
FD	−0.455**	−0.463*	−0.339*	−0.084	−0.280
	(0.203)	(0.243)	(0.192)	(0.258)	(0.233)
CONSTANT	6.794***	4.599***	4.426***	5.801***	5.806***
	(0.982)	(1.178)	(0.930)	(1.249)	(1.129)
OBSERVATIONS	170	170	170	170	170

Standard errors in parentheses.

***p < 0.01, **p < 0.05, *p < 0.1.

Another interesting result is the negative relationship found in some specifications between geopolitical risk and PM2.5 exposure. One possible way to understand this result is that during periods of increased instability, the level of economic activity, industrial production, transportation, and energy consumption may be temporarily reduced, thus lowering the level of particulate matter emitted in the short run. This would be in line with the notion that higher levels of macro-level uncertainty may reduce pollution-generating activities in the short run, even if these activities are still damaging to the environment in the long run.

The interaction term CO2GPRC, which captures the combined effect of CO2 emissions and geopolitical risk on PM2.5, consistently shows a positive and highly significant coefficient (approximately 0.001***, implying a very small but statistically significant positive interaction). This suggests that while geopolitical itself might depress PM2.5 in some contexts, their interplay with existing CO2 emissions levels could still contribute to air pollution. Again, this result must be interpreted with some caution since the present study did not explicitly model the output/sectoral composition channel through which the relationship may be transmitted, and hence the result speaks more to the existence of a possible channel than to the identification of the channel itself.

Per capita income consistently demonstrates a negative and highly significant association with PM2.5 (−0.486 to −0.617), reinforcing the notion that economic development, potentially through technological advancements and stricter environmental regulations, tends to improve air quality. Population, however, shows a mixed effect in pooled OLS: an initial negative and significant correlation (−0.102) in the basic model, becoming insignificant or slightly positive in more comprehensive models. Foreign Direct Investment exhibits a negative and significant relationship with PM2.5 (−0.194 to −0.197), suggesting that FDI may contribute to cleaner technologies or practices. Conversely, trade openness has a consistent positive and significant impact on PM2.5 (0.223 to 0.267), potentially due to increased industrial activity or transportation. Financial development, in the most comprehensive pooled model, is not statistically significant.

The results of the QRPD model for PM2.5, as presented in Table 7, reveal nuanced effects across the distribution of air pollution. The positive impact of CO2 emissions on PM2.5 generally strengthens at higher quantiles (e.g., from 0.218 at Q-0.10 to 0.454 at Q-0.90 in the simplest QRPD model), indicating that in environments already facing high carbon emission levels, further increases in CO2 emissions lead to disproportionately higher PM2.5 concentrations. Conversely, the negative effect of geopolitical risk on PM2.5 remains robust and significant across most quantiles, often being stronger at lower and middle quantiles. This could imply that its pollution-dampening effect, possibly via economic slowdown, is more pronounced when air quality is moderately bad. The interaction term of CO2 emissions and geopolitical risk remains consistently positive and significant across almost all quantiles in the QRPD models, reaffirming that despite the geopolitical direct depressive effect on PM2.5, its interaction with CO2 emissions exacerbates air pollution, highlighting a critical amplifying mechanism.

The ameliorating effect of per capita income on PM2.5 is consistently significant and negative across all quantiles, underscoring the broad benefits of economic development for air quality. The influence of population is less consistent across quantiles, sometimes negative and significant at lower quantiles, but becoming insignificant or weakly negative at higher ones. Foreign Direct Investment also shows a largely negative and significant effect, particularly at middle quantiles, suggesting its role in promoting cleaner production as pollution levels rise. Trade openness consistently contributes positively to PM2.5, especially at lower and middle quantiles. Finally, financial development generally shows a negative influence on PM2.5 at lower and middle quantiles, but its significance wanes at higher quantiles in the most comprehensive QRPD model.

In conclusion, both pooled OLS and QRPD results confirm that CO2 emissions significantly increase the threat to public health through PM2.5 pollution. While geopolitical risk can, surprisingly, be associated with lower PM2.5 in some average scenarios (possibly due to economic disruption), its interaction with CO2 emissions suggests an exacerbating effect on air quality. Economic development consistently improves air quality, while trade openness tends to worsen it. The quantile regressions uniquely highlight how the intensity of these relationships varies across different levels of air pollution, offering critical insights for targeted public health and environmental policy interventions in the BRICS countries.

### Life expectancy

The regression analysis for life expectancy, a key public health indicator, reveals critical insights into its drivers in BRICS nations. Across both pooled OLS results shown in Table 6 and QRPD regression results as presented in Table 8, CO2 emissions consistently show a negative and statistically significant impact on life expectancy. This suggests that increased environmental quality, as proxied by higher CO2 emissions levels, generally corresponds to a reduction in life expectancy, directly linking environmental quality to public health outcomes.

**Table 8:** Quantile regression estimators: results (dep var.: life expectancy).

	(1)	(2)	(3)	(4)	(5)
Equation 7					
LNCO2	−0.029***	−0.022***	−0.018***	−0.003	−0.030***
	(0.008)	(0.006)	(0.006)	(0.007)	(0.004)
GPRC	0.068***	0.047***	0.030***	0.017	0.034***
	(0.014)	(0.011)	(0.010)	(0.012)	(0.007)
CO2GPRC	−0.000***	−0.000***	−0.000***	−0.000***	−0.000***
	(0.000)	(0.000)	(0.000)	(0.000)	(0.000)
LNPOP	0.132***	0.109***	0.096***	0.072***	0.083***
	(0.007)	(0.006)	(0.005)	(0.007)	(0.003)
LNGDPPC	0.140***	0.125***	0.125***	0.113***	0.091***
	(0.008)	(0.006)	(0.006)	(0.007)	(0.004)
CONSTANT	0.640***	1.176***	1.444***	1.936***	2.095***
	(0.169)	(0.134)	(0.119)	(0.151)	(0.080)
Equation 8					
LNCO2	−0.046***	−0.043***	−0.033***	−0.003	−0.011*
	(0.008)	(0.011)	(0.010)	(0.012)	(0.006)
GPRC	0.113***	0.089***	0.056***	0.031*	0.029***
	(0.013)	(0.017)	(0.015)	(0.018)	(0.008)
CO2GPRC	−0.000***	−0.000***	−0.000***	−0.000***	−0.000***
	(0.000)	(0.000)	(0.000)	(0.000)	(0.000)
LNPOP	0.134***	0.121***	0.101***	0.066***	0.067***
	(0.006)	(0.009)	(0.008)	(0.009)	(0.004)
LNGDPPC	0.135***	0.124***	0.123***	0.098***	0.091***
	(0.006)	(0.008)	(0.007)	(0.008)	(0.004)
LNFDI	0.033***	0.022**	0.012	0.011	0.008*
	(0.007)	(0.009)	(0.008)	(0.010)	(0.004)
LNTO	−0.004	0.010	0.014	−0.019	−0.021***
	(0.011)	(0.015)	(0.013)	(0.016)	(0.007)
CONSTANT	0.731***	1.031***	1.378***	2.213***	2.333***
	(0.144)	(0.198)	(0.174)	(0.207)	(0.096)
Equation 9					
LNCO2	−0.047***	−0.034***	−0.030***	0.006	−0.007
	(0.010)	(0.012)	(0.011)	(0.013)	(0.005)
GPRC	0.113***	0.088***	0.056***	0.025	0.028***
	(0.014)	(0.017)	(0.016)	(0.018)	(0.007)
CO2GPRC	−0.000***	−0.000***	−0.000***	−0.000***	−0.000***
	(0.000)	(0.000)	(0.000)	(0.000)	(0.000)
LNPOP	0.135***	0.117***	0.097***	0.060***	0.064***
	(0.008)	(0.010)	(0.009)	(0.011)	(0.004)
LNGDPPC	0.135***	0.125***	0.117***	0.094***	0.086***
	(0.008)	(0.009)	(0.009)	(0.010)	(0.004)
LNFDI	0.031***	0.020**	0.013	0.010	0.007*
	(0.008)	(0.009)	(0.008)	(0.010)	(0.004)
LNTO	−0.004	−0.004	0.010	−0.023	−0.026***
	(0.014)	(0.017)	(0.016)	(0.018)	(0.007)
FD	0.013	0.069	0.048	0.035	0.016
	(0.040)	(0.048)	(0.044)	(0.051)	(0.020)
CONSTANT	0.712***	1.064***	1.480***	2.311***	2.426***
	(0.194)	(0.231)	(0.213)	(0.248)	(0.096)
OBSERVATIONS	170	170	170	170	170

Standard errors in parentheses.

***p < 0.01, **p < 0.05, *p < 0.1.

The positive relationship found between geopolitical risk and life expectancy should also be interpreted with caution. The coefficient on this variable should not be interpreted as suggesting that instability in geopolitical risks is, in itself, good for health. The finding should be seen as possibly reflecting aggregation effects, health trend effects, adaptation in the public sector, or effects of omitted structural variables that are not captured well in this macro-panel framework. There is a wide range of long-term socioeconomic, healthcare, institutional, and demographic determinants that influence life expectancy, and this variable’s response to geopolitical risks is not necessarily similar to that found with more proximal exposure to environmental risks, such as PM2.5. The finding should be seen as a conditional relationship within this specific statistical model rather than as reflecting a causal, direct, beneficial effect of geopolitical risks.

Population and per capita income consistently display a positive and significant association with life expectancy across most models and quantiles, underscoring the benefits of demographic stability and economic prosperity for public health. Other variables like FDI, trade openness, and financial development show varying or less consistent effects. These results highlight the essentials for comprehensive policy strategies that address environmental quality and geopolitical stability to bolster public health outcomes in these rapidly evolving economies.

### Fixed effects (within) estimator with Driscoll-Kraay standard errors (for robustness)

For robustness of the results and to address method dependence and small-N, large-T panel structure, the study conducts an alternative robustness estimation using Driscoll–Kraay standard errors, which explicitly correct for heteroskedasticity, serial correlation, and cross-sectional dependence. This estimator is particularly appropriate for BRICS panels (N = 5, T = 34) and confirms that the main results are not driven by estimation method choice. The results are depicted in (Appendix A) Hence, the key findings are stable beyond pooled OLS and quantile specifications.

## Discussion and policy implications

The in-depth study of geopolitical risk, environmental quality, and public health in the BRICS countries demonstrates how contrary yet connected these aspects of the landscape are. Evidence from the analyses strongly suggests that geopolitical risk significantly contributes to environmental quality; it robustly correlates with CO2 emissions positively (see Table 8).

This means that with increasing geopolitical risk, policy focus may be diverted from protecting the environment and worsening CO2 emissions due to issues such as fossil fuel-based energy security and weak regulation and enforcement. Concurrently, population and per capita income growth are related to greater CO2 emissions, further revealing the development-related pressure on environmental quality being faced by these rapidly expanding economies. While FDI and trade openness generally push up emissions, one important counterbalancing variable is financial development, which is negatively associated with CO2 emissions, suggesting that it creates a pathway toward greener transitions.

Unmistakably in public health terms, PM2.5 air pollution is directly and strongly linked to environmental quality. Thus, confirming the direct public health fallout of unsustainable environmental practices. The interaction of geopolitical risk with PM2.5 is quite intricate; on the one hand, pooled analyses would suggest a direct negative correlation (likely to reflect economic slowdowns during instability); however, on the other, the interaction term between environmental quality and geopolitical risk always indicates that geopolitical risk exacerbates the negative impacts of environmental quality on public health. Hence, we have one critical compounding threat to public health; emissions grow in the face of instability, and those already overburdened environments are then compounded by turbulence in the geopolitical sphere. Also, this study has found that while per capita GDP tends to foster clean air, trade liberalization is often at odds with public health (PM2.5).

Life Expectancy is a strong indicator of the overall public health. The study shows that increased CO2 emissions reduce life expectancy, thus accentuating the aggregated health impacts of environmental unsustainability. The interaction between CO2 emissions and geopolitical risk tends to aggravate this negative association on the longevity scale. Population size growth and per capita GDP are usually manifested in a positive relation to life expectancy and thus demonstrate benefits of development, which usually suffer from increasing environmental burdens, especially when compounded by geopolitical tensions. The generalized quantile regression results show that these relationships are not the same for different environmental conditions and health outcomes, showing heterogeneous effects requiring more considered policy responses.

The insights from this study suggest coordinated policy efforts within the BRICS countries. It is not sufficient for policymakers to treat geopolitical stability, environmental quality, and public health as separate policy initiatives; they actually need to be viewed as deeply intertwined. Therefore, the policymakers in BRICS must address geopolitical risks strategically if environmental quality and public health are to be reached. Some important steps could include promoting diplomatic solutions to regional tensions, diversifying energy sources to diminish dependence on carbon-abundant fuels and supporting transparency in governance that can weather geopolitical shocks. It is equally important to utilize financial development to mobilize investment into green technologies and infrastructure. This finding has vital implication as it may help to partially offset environmental pressure even under geopolitical stress. One potential mechanism for this could be that more developed financial systems facilitate access to capital for cleaner technologies, energy efficiency upgrades, and environmental infrastructure, thus offsetting the carbon footprint of economic activity. In addition, more developed financial markets may allow firms to adjust to external pressures more flexibly, without necessarily relying on pollution-intensive adjustment paths. In this respect, financial development may act as a mechanism to reduce the environmental impact of geopolitical instability. The heterogeneous impacts indicated by quantile regression also strengthen the need for interventions, as the most efficient policy interventions for improved environmental and health outcomes will likely differ with the specific stage of development of a country and the level of pollution or health challenges that it currently has.

## Conclusion

The empirical context of this study is specifically limited to the BRICS countries due to the significant population size, rising environmental pressures, and high geopolitical tensions faced by them. However, this also limits the generalizability of the findings. It is suggested that the results should be viewed as indicative of the relationship between the variables within the context of emerging economies and not necessarily as applicable across all developing or developed nations worldwide.

This paper attempted to address an under-explored and complex relationship between geopolitical risk, environmental deterioration, and public health, in the context of BRICS countries. These countries are in rapid development and exert heavy environmental footprints and are prone to environmental and geopolitical risks. This presents an ideal setting to understand how geopolitical risks may cause rippling effects through environmental quality and implications for public health. The research design is underpinned by an econometric framework of high validity employing pooled and quantile regressions panel dataset models to study long panels in order to establish long-term average effects as well as heterogeneous impacts across levels of geopolitical risks, CO2 emissions, and health outcomes. This was the basis of insight into the rationale of complex causations and interdependencies among the variables.

The most important insight from our study shows an intertwined relationship among variables. To begin with, a significant and consistent factor of CO2 emissions is geopolitical risk. This indicates that a period of instability is not merely an economic question but rather one of environment, which negatively impacts fossil fuel dependency and may induce relaxing curbs on green initiatives. Second, the findings clearly indicate that higher CO2 emissions are associated with poorer air quality, as evidenced by increasing levels of PM2.5 concentrations thereby linking environmental quality as an indirect way of threatening human health. Lastly, and most importantly, life expectancy analysis implies that these challenges perpetuate each other. Economic growth and increased population can impact life expectancy through improved standards of living. However, advances in life expectancy are thwarted by the negative effects of CO2 emissions. Our findings explicitly show that interactions between CO2 emissions and geopolitical risks further worsen life expectancy. Hence arises a powerful and dangerous interaction: one that enlarges the negative impact of CO2 emissions and environmental quality.

In essence, the study provides compelling evidence that geopolitical risks pose a direct threat to the environmental and public health of the BRICS nations. The results underscore the necessity for policymakers to integrate environmental and public health considerations into their geopolitical strategies. The major takeaway is that the development of finance helps alleviate the pressure on the environment during geopolitical crises. This is because, with the help of finance, the carbon intensity of the economy is decreased, allowing the economy to become more efficient without the need to implement harmful practices. The buffer effect of finance is what helps the economy become resilient. These results give policymakers a clear approach: strengthen financial institutions to keep up environmental progress even when times are unstable. The direct impact of FD on PM2.5 and life expectancy is weaker and less consistent. This implies that FD alone is not sufficient rather effective policy should integrate FD with targeted health and environmental interventions to enhance public health protection against geopolitical shocks. This is no longer a matter of siloed policy domains, but a holistic challenge where instability can compromise human and planetary health simultaneously.

This study should be viewed in the context of the following limitations. Firstly, the empirical strategy does not fully address endogeneity concerns, and it is possible that omitted institutional or structural factors are affecting the results. Secondly, the empirical strategy does not explicitly address dynamic persistence, particularly in the context of environmental or long-run health outcomes. Thirdly, the BRICS-centric sample is useful for analytical purposes but restricts the external validity of the results for these five countries. Finally, certain channels of transmission, such as military expenditure, energy composition, and growth mediation, are not separately identified in this framework. While these limitations do not detract from the policy or empirical interest of the results, they caution that the results should be viewed as robust conditional associations that require further exploration.

There are a number of ways that future research can extend this study. One is that future studies could attempt to estimate dynamic panel models that control more effectively for persistence and lagged adjustment effects. Another is that future studies could extend this analysis to more countries beyond the BRICS countries in order to see if the relationships found generalize across different contexts. Finally, future studies could explore specific channels through which this relationship is mediated by military spending, energy system dependence, restructuring effects, and GDP growth.

## Data Availability

The raw data supporting the conclusions of this article will be made available by the authors, without undue reservation.
