# Supplementary material for: Cascading effects of geopolitical risk on environmental quality and public health in BRICS
Source: Front Public Health. 2026 Apr 21;14:1703933. doi: 10.3389/fpubh.2026.1703933 (PMC13139357; doi:10.3389/fpubh.2026.1703933)
Supplement: Supplementary file 1 [file Data_Sheet_1.docx]

**Appendix A**

**Fixed Effects (within) estimator with Driscoll-Kraay Standard Errors**

| VARIABLES | lnco2 | lnpm25 | lnle |
| --- | --- | --- | --- |
|  |  |  |  |
| gprc | 0.207*** | 0.208** | -0.038*** |
|  | (0.079) | (0.094) | (0.012) |
| lnpop | 1.161*** | -0.103 | 0.371*** |
|  | (0.293) | (0.149) | (0.105) |
| lngdppc | 0.623*** | -0.499*** | 0.103*** |
|  | (0.041) | (0.066) | (0.018) |
| lnfdi | 0.181*** | -0.431*** | 0.053*** |
|  | (0.031) | (0.043) | (0.02) |
| lnto | 0.138** | -0.003 | -0.024*** |
|  | (0.067) | (0.062) | (0.008) |
| fd | -0.334*** | -0.796*** | -0.042 |
|  | (0.103) | (0.114) | (0.034) |
| lnco2 |  | 0.681*** | -0.069* |
|  |  | (0.126) | (0.039) |
| co2gprc |  | 0.001*** | -0.000*** |
|  |  | (0.0001) | (0.000) |
| Constant | -21.045*** | 5.151** | -3.284* |
|  | (5.204) | (2.394) | (1.892) |
|  |  |  |  |
| Observations | 170 | 170 | 170 |
| Number of groups | 5 | 5 | 5 |

Standard errors in parentheses

*** p<0.01, ** p<0.05, * p<0.1

**Appendix B**

**Dumitrescu Hurlin causality tests**

H_0_: Geopolitical Risk does not Granger-cause Environmental Quality.

H_1_: Geopolitical Risk does Granger-cause Environmental Quality.

$$\bar{Z} = 5.109$$

$$p = 0.000$$

***Decision Reject H_0._***

H0: Environmental Quality does not Granger-cause Geopolitical Risk.

H1: Environmental Quality does Granger-cause Geopolitical Risk.

$$\bar{Z} = 1.844,$$

$$p = 0.890$$

***Decision: Do not Reject H_0._***

**Pesaran (2004) cross-sectional dependence test**

CD = 0.900,

*p* = 0.368
